# The Association Between Selenium, Selenoprotein P (SEPP1), Fluid Intelligence, and Exercise in the UK Biobank Cohort

**DOI:** 10.7759/cureus.25353

**Published:** 2022-05-26

**Authors:** Steven Lehrer, Peter H Rheinstein

**Affiliations:** 1 Radiation Oncology, Icahn School of Medicine at Mount Sinai, New York City, USA; 2 Family Medicine, Severn Health Solutions, Severna Park, USA

**Keywords:** nutrition, exercise, cognition, fluid intelligence, selenium

## Abstract

Background: In the mouse hippocampus, exercise boosts neurogenesis. Increased levels of the selenium transport protein selenoprotein P (SEPP1) in the serum of exercised animals may contribute to the impact of exercise. SEPP1 is a protein that aids in the delivery of selenium to the brain. The effect of exercise on mouse brain precursor cell proliferation was diminished when SEPP1 or its receptor were genetically depleted. Selenium supplementation in the diet had the same effect as exercise in reducing some of the cognitive impairments associated with aging.

Methods: In the current analysis, we sought to determine the association of selenium, the SEPP1 gene, fluid intelligence, and exercise in the UK Biobank Cohort. We analyzed SEPP1 single nucleotide polymorphism (SNP) rs7579, a single nucleotide variation (SNV), position chr5:42800706, C > T, minor allele frequency T = 0.281. Its consequence is a 3’- UTR variant. The 3′-UTR contains regulatory regions that post-transcriptionally influence gene expression and is responsible for selenoprotein synthesis. SNP rs7579 has been implicated in multiple forms of cancer. The univariate general linear model of SPSS (IBM Corp., Armonk, NY) was used to rule out the effects of age, years of education, and vigorous activity on fluid intelligence score, with fluid intelligence score as the dependent variable, rs7579 genotype, and selenium supplements as fixed factors, and age, years of education, and vigorous activity as covariates.

Results: The effect of rs7579 genotype on fluid intelligence score was insignificant (p = 0.702). The effect of selenium supplements on fluid intelligence score was insignificant (p = 0.107). The interaction of rs7579 genotype and selenium supplements was insignificant (p = 0.911) and unrelated to the significant effects of age (p < 0.001), years of education (p < 0.001), and vigorous activity (p < 0.001) on fluid intelligence score.

Conclusion: Our multivariate analysis of SEPP1 genotype, selenium supplement use, and fluid intelligence scores is consistent with the negligible effect selenium supplements seem to have on cognition. Selenium is found in nuts, dairy products, and grains. These foods can provide sufficient selenium for health. Selenium supplements are not recommended.

## Introduction

Adult hippocampal neurogenesis is an evolutionarily conserved process that supplies the brain with structural plasticity enabling good learning and memory. Although strong evidence indicates that adult hippocampal neurogenesis occurs in humans and may underpin cognitive processes important for adaptive behavior, we rely on animal studies to learn how activity-dependent regulation of adult neurogenesis occurs at a cellular level.

In the mouse hippocampus, for example, exercise boosts neurogenesis. Leiter et al. looked for proteins that increased in quantity when mice ran on a treadmill [1]. They report that increased levels of the selenium transport protein selenoprotein P (SEPP1) in the serum of exercised animals may contribute to the impact of exercise. SEPP1 is a protein that aids in the delivery of selenium to the brain. The effect of exercise on brain precursor cell proliferation was diminished when SEPP1 or its receptor were genetically depleted. Selenium supplementation in the diet had the same effect as exercise in reducing some of the cognitive impairments associated with aging.

In the current analysis, we sought to determine the association of selenium, the SEPP1 gene, cognition, and exercise in the UK Biobank (UKBB) cohort. Valuable insights are provided by this type of genetic data mining/methodology. Many websites, including 23andMe (www.23andme.com), are discovering connections between genetics, health, and disease.

## Materials and methods

The UK Biobank is a large prospective observational study of men and women. Participants were recruited from across 22 centers located throughout England, Wales, and Scotland between 2006 and 2010 and continue to be longitudinally followed for the capture of subsequent health events [2]. This methodology is like that of the ongoing Framingham Heart Study [3], with the exception that the UKB program collects postmortem samples, which Framingham did not.

Our UK Biobank application was approved as UKB project 57245 (S.L., P.H.R.). Our analysis included all subjects with physical activity assessment, selenium supplement use, fluid intelligence score, and genotype of SEPP1 single nucleotide polymorphism (SNP) rs7579.

SEPP1 SNP rs7579 is a single nucleotide variation (SNV), position chr5:42800706, C > T, minor allele frequency T = 0.281. Its consequence is a 3’- UTR variant. The three prime untranslated region (3′-UTR) is the section of messenger RNA (mRNA) that immediately follows the translation termination codon. The 3′-UTR contains regulatory regions that post-transcriptionally influence gene expression and is responsible for selenoprotein synthesis. SNP rs7579 has been implicated in multiple forms of cancer [4,5].

The fluid intelligence score is from the UK Biobank data field 20191. The score is a simple unweighted sum of the number of correct answers given to the 13 fluid intelligence questions. Participants who did not answer all the questions within the allotted two-minute limit were scored as zero for each of the unattempted questions. Despite the brief, non-standard nature of the UK Biobank cognitive tests, the scores have shown substantial concurrent validity and test-retest reliability [6].

The duration of intense activity (minutes per day) was measured using a questionnaire from UK Biobank category 100054 data field 914. Selenium use is from UK Biobank data field 6179, mineral and other dietary supplements.

Data processing was performed on Minerva, a Linux mainframe with Centos 7.6, at the Icahn School of Medicine at Mount Sinai. We used PLINK, a whole-genome association analysis toolset, to process the UKB chromosome files [7] and the UK Biobank Data Parser (ukbb parser), a python-based package that allows easy interfacing with the large UK Biobank dataset [8]. SPSS version 26 (IBM Corp., Armonk, NY) was used for statistical analysis.

## Results

We analyzed data from 120,803 subjects. Demographic data are in Table 1.

**Table 1:** Demographics of subjects studied.

n	120803			
age	56.5	±	8	years
sex	54%			female
race	95%			white
education	14.6	±	5	years

**Figure 1:**
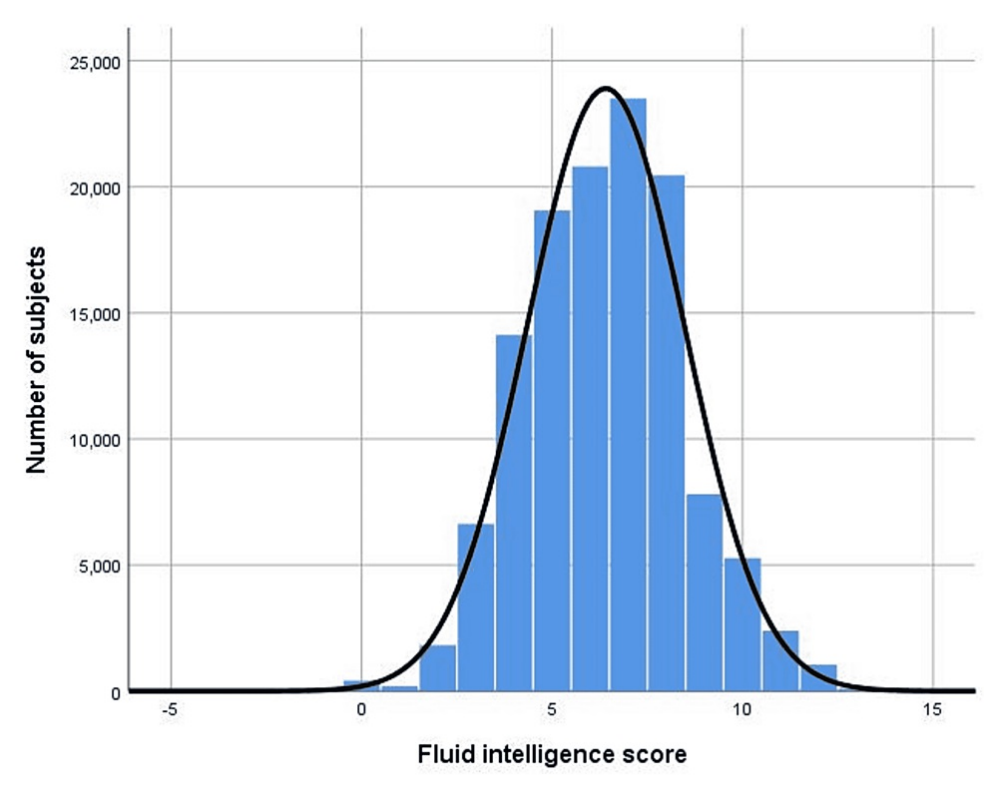
Distribution of fluid intelligence scores in 120,803 subjects with superimposed normal curve.

**Figure 2:**
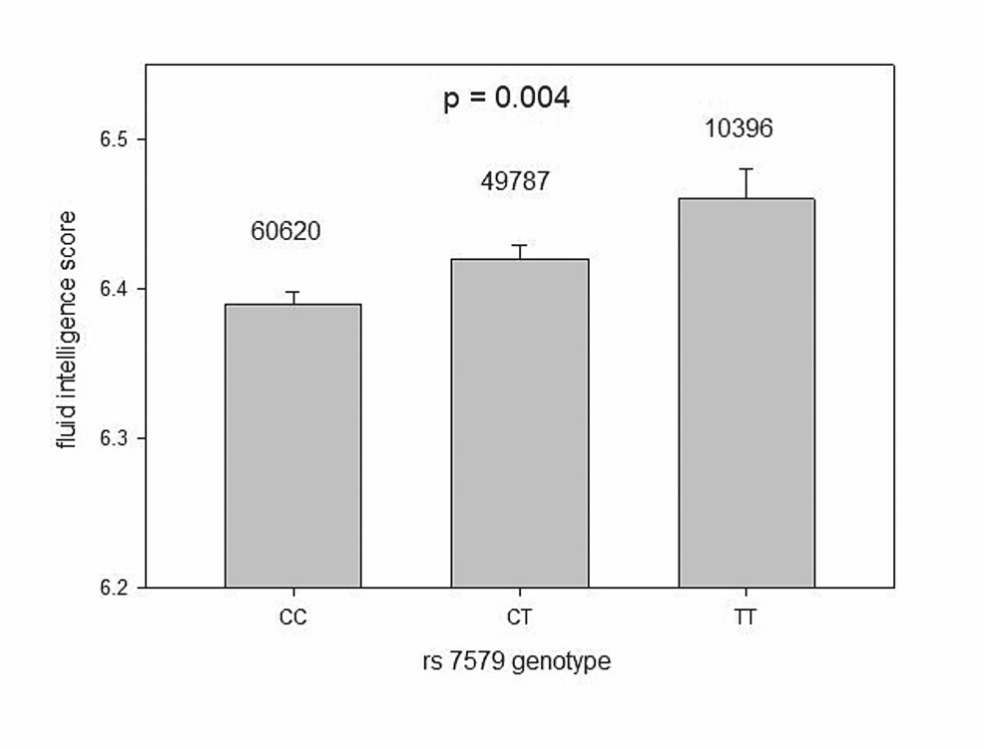
Fluid intelligence score by SNP rs7579 genotypes CC, CT, and TT (mean + SEM). Number of subjects in each group above corresponding error bar. Because of the large sample size, the tiny difference in fluid intelligence score (effect size) by each genotype, while statistically significant, is probably clinically meaningless. The average fluid intelligence score was 6.41 with a difference of 0.03 between each genotype. SNP: single nucleotide polymorphism; SEM: standard error of the mean.

The distribution of fluid intelligence scores in 120,803 subjects with superimposed normal curve is in Figure 1. The fluid intelligence score by SEPP1 SNP rs7579 genotypes is in Figure 2. Genotype TT had the highest score. Because of the large sample size, the tiny difference in fluid intelligence score (effect size) by each genotype, while statistically significant (p = 0.004 one way ANOVA), is probably clinically meaningless [9]. The average fluid intelligence score was 6.41 with a difference of 0.03 between each genotype; 639 carriers of the minor allele T had taken selenium supplements.

**Figure 3:**
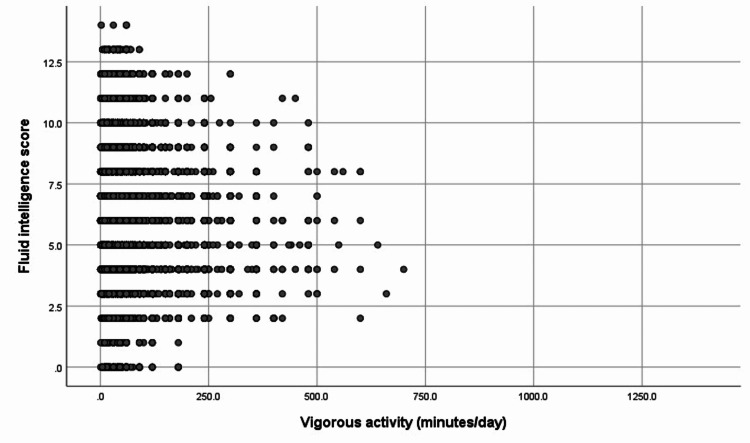
Vigorous activity (minutes/day) versus fluid intelligence score. The beneficial effect of vigorous activity appears to peak at about 500 minutes per day, which is heavy non-stop manual labor for more than eight hours.

Vigorous activity (minutes/day) versus fluid intelligence score is in Figure 3. The beneficial effect of vigorous activity appears to peak at about 500 minutes per day, which is heavy non-stop manual labor for more than eight hours. Physical exercise or physical activities are known to promote brain and cognitive vitality well into older adulthood [10].

A total of 42,337 subjects who had not taken selenium had fluid intelligence score 6.07 ± 1.95 (mean ± SD). A total of 142 subjects who had taken selenium had fluid intelligence score 6.53 ± 2.09 (p = 0.005, two-tail t-test). Because of the large sample size, the tiny increase in fluid intelligence score (effect size) with selenium is probably clinically meaningless.

The univariate general linear model of SPSS was used to rule out the effects of age, years of education, and vigorous activity on fluid intelligence score, with fluid intelligence score as the dependent variable, rs7579 genotype, and selenium supplements as fixed factors, and age, years of education, and vigorous activity as covariates. The effect of rs7579 genotype was insignificant (p = 0.702), the effect of selenium supplements was insignificant (p = 0.107), the interaction of rs7579 genotype and selenium supplements was insignificant (p = 0.911) and unrelated to the significant effects of age (p < 0.001), years of education (p < 0.001), and vigorous activity (p < 0.001).

## Discussion

Selenium is a trace element found in many foods and added to others, while sold as a dietary supplement. Selenium is a component of more than two dozen selenoproteins, which play important roles in reproduction, thyroid hormone metabolism, DNA synthesis, and protection from oxidative damage and infection in humans [11].

The first concrete evidence that selenium plays a specialized role in the brain came from a study of children with intractable seizures who were shown to have poor glutathione peroxidase activity and who improved clinically after taking selenium supplements [12]. The concentration of selenium in the blood decreases with age, and selenium deficiency may be linked to age-related impairments in brain function, presumably due to a reduction in selenium's antioxidant action [13,14].

However, observational studies have yielded conflicting results [15]. Subjects with lower plasma selenium levels at baseline were more likely to develop cognitive impairment over time in two major investigations, although it is unclear if the participants in these studies were selenium deficient [13,16]. There was no link between serum selenium levels (which ranged from lower than 11.3 to higher than 13.5 mcg/dL) and memory test scores in a review of NHANES data on 4,809 older persons in the United States [17]. Our multivariate analysis of fluid intelligence scores and SEPP1 SNP rs7579 genotypes suggests that SEPP1 selenoprotein does not significantly affect fluid intelligence scores.

Researchers investigated whether elderly people who took a selenium-rich antioxidant supplement had a lower risk of cognitive decline. Higher episodic memory and semantic fluency test scores were related to daily intake of 120 mg ascorbic acid, 30 mg vitamin E, 6 mg beta-carotene, 100 mcg selenium, and 20 mg zinc over eight years [18]. The independent contribution of selenium to the reported effects in this investigation cannot be determined.

The present clinical evidence is insufficient to evaluate if selenium supplements can prevent Alzheimer's disease, according to a systematic review that included nine placebo-controlled studies [15]. Our multivariate analysis of selenium supplement use and fluid intelligence scores, presented above, is consistent with the negligible effect that these supplements seem to have on cognition.

The fact that SEPP1 is involved in mouse neurogenesis, but probably not human, as UKBB data above suggest, is hardly surprising. A genome analysis, by 20 institutions from six countries, showed that humans, rats, and mice have about the same number of genes. But it also revealed that humans and rodents descended from a common ancestor about 80 million years ago, with rats and mice diverging between 12 and 24 million years ago [19]. Thus, over millions of years, SEPP1 may have developed vastly different functions in mice and humans [20].

The federal government’s 2020-2025 Dietary Guidelines for Americans notes that “Because foods provide an array of nutrients and other components that have benefits for health, nutritional needs should be met primarily through foods. … In some cases, fortified foods and dietary supplements are useful when it is not possible otherwise to meet needs for one or more nutrients (e.g., during specific life stages such as pregnancy).”

## Conclusions

Nuts, dairy products, and cereals all contain selenium. These foods contain enough selenium to be healthy. Supplementing with selenium is not advised.
